# Effects of Injections of Monocytes, Platelet-Rich Plasma, and Hyaluronic Acid in Adults with Knee Osteoarthritis: An Observational Study

**DOI:** 10.3390/jfmk10020104

**Published:** 2025-03-26

**Authors:** Rita Chiaramonte, Salvatore Caramma, Enrico Buccheri, Patrizia Finocchiaro, Umile Giuseppe Longo, Antonio Ammendolia, Alessandro de Sire, Michele Vecchio

**Affiliations:** 1 Department of Biomedical and Biotechnological Sciences, University of Catania, 95123 Catania, Italy; ritachiaramd@gmail.com (R.C.); enrico.buccheri@gmail.com (E.B.); patry.finocchiaro@gmail.com (P.F.); michele.vecchio@unict.it (M.V.); 2 Department of Pain Management, Policlinico-San Marco Hospital, 95123 Catania, Italy; salvacaramma@tiscali.it; 3 Fondazione Policlinico Universitario Campus Bio-Medico, Via Alvaro del Portillo, 200, 00128 Roma, Italy; g.longo@policlinicocampus.it; 4 Research Unit of Orthopaedic and Trauma Surgery, Department of Medicine and Surgery, Università Campus Bio-Medico di Roma, Via Alvaro del Portillo, 21, 00128 Roma, Italy; 5 Physical and Rehabilitative Medicine, Department of Medical and Surgical Sciences, University of Catanzaro “Magna Graecia”, 88100 Catanzaro, Italy; ammendolia@unicz.it; 6 Research Center on Musculoskeletal Health, MusculoSkeletalHealth@UMG, University of Catanzaro “Magna Graecia”, 88100 Catanzaro, Italy; 7 Rehabilitation Unit, AOU Policlinico G. Rodolico-San Marco, 95123 Catania, Italy

**Keywords:** intra-articular injections, hyaluronic acid, platelet-rich plasma, monocytes, rehabilitation, knee osteoarthritis

## Abstract

**Background:** Knee osteoarthritis (OA) is a prevalent condition among adults that leads to knee joint pain and dysfunction. Over the past two decades, local intra-articular knee injection therapy has gained popularity due to the advent of platelet-rich plasma (PRP), hyaluronic acid (HA), and the novel peripheral blood-derived mononuclear cells (PBMNCs). This study aimed to compare the therapeutic efficacy of intra-articular injections of PBMNCs, HA, and PRP combined with hyaluronic acid (PRP-HA) for treating degenerative knee OA classified as stages II and III, according to the Kellgren and Lawrence (KL) scale. **Methods:** This retrospective observational study involved adults with moderate-to-moderately severe knee OA treated at the University Hospital of Catania, Italy. The subjects were divided into three groups and treated with intra-articular injections of HA, PRP-HA, or PBMNCs. The outcome measures assessed were as follows: the Numerical Rating Scale, the Western Ontario and McMaster Universities Arthritis Index, the Timed Up and Go, the International Knee Documentation Committee score, a 10-meter walking test, and the Physical score and the Mental score on the SF-12. This study included a total of 46 adults, 30 females and 16 males, with a mean age of 63.7 ± 10.9 years. **Results:** HA, PRP-HA, and PBMNCs demonstrated comparable effectiveness for improving the NRS score and all the other outcomes at 6 months. Additionally, PRP-HA and PBMNCs also enhanced knee flexion and the International Knee Documentation Committee score. However, none of the three treatments led to a significant improvement on the 10-meter walking test. No serious adverse effects were reported. **Conclusions:** In this study, injections of HA, PRP-HA, and PBMNCs all demonstrated positive outcomes for up to 6 months post-treatment in the subjects suffering from knee OA.

## 1. Introduction

Knee osteoarthritis (OA) is a common condition in the adult population, leading to pain and the functional impairment of the knee joint. Indeed, osteoarthritis leads to reduced mobility, with related complications, such as loss of bone mass, increased risk of fractures, and diminished quality of life of the affected individuals [1,2,3,4].

The causes of OA include articular cartilage lesions, with poor healing and regeneration potential, because cartilage does not contain vessels or nerves. In addition, inflammatory processes are not adequately resolved at the cartilage level, and biomechanical, metabolic, and biological changes related to aging or trauma ultimately lead to the degeneration of articular cartilage, which defines OA [5].

Factors such as repetitive loads and lifestyle choices, including alcohol consumption, tobacco use, and overweight or obesity, are recognized as risk factors for OA [6], just as climatic conditions seem to play a role in the resurgence of pain, although further studies on the subject are still needed [7]. Anyway, age represents the most important risk factor, as it favors the progressive loss of homeostatic responses and the consequent cartilage degeneration. The consequence is that OA occurs more in subjects older than 60–69 years, while it is far less frequent in younger individuals [8].

Its conservative treatment consists mainly of therapeutic exercise, and represents the first line of treatment for OA, given its modulating function on various biological molecules [9]; nutraceutical supplementation has also been shown to be an adjuvant strategy in the management of OA [10,11].

The prevalence of knee OA is 16% in males [8] and its incidence rises linearly within the 50–80 age bracket [6], underscoring its status as a leading cause of disability among the elderly [8]. This condition is often associated with a significantly reduced quality of life [8]. Plain radiographs serve as a cornerstone in the diagnosis of OA. The Kellgren and Lawrence (KL) classification system, first formalized for OA in 1957, remains in widespread use, especially in the context of knee OA [12]. This system offers a five-grade classification scale that reflects the progressive severity of OA; specifically, grade 0 indicates no OA, and grade 4 represents severe OA [12]. A comprehensive assessment of the disease state is necessary for a definitive diagnosis of OA, beyond the use of the KL system. It is recommended that the KL system be complemented by a thorough clinical evaluation, including a detailed medical history, physical examination, and radiographic imaging to diagnose knee OA [13].

In this scenario, the conservative treatment for subjects with knee OA might consist not only of oral anti-inflammatory drugs, nutraceuticals, and physical therapy, but also, as a second line treatment, intra-articular injections of corticosteroids, hyaluronic acid (HA), platelet-rich plasma (PRP), and oxygen–ozone therapy [9,14,15,16,17].

In the field of intra-articular injection therapy, treatments such as bone marrow-derived mesenchymal stem cell (BM-MSC)-enriched plasma, peripheral blood-derived mononuclear cells (PBMNCs), and PRP injections have garnered significant attention due to their trophic support of other cells and their ability to secrete bioactive factors [18,19,20,21,22,23].

PRP contains growth factors, including platelet-derived growth factors like growth factor β, insulin-like growth factor 1, and many others, that play an important role in cartilage homeostasis and cartilage regeneration [5]. BM-MSCs appear to delay the progression of cartilage degeneration and prevent chondrocyte apoptosis through a paracrine effect, thereby relieving pain and enhancing joint function [24]. An alternative approach involves viscosupplementation with HA [25,26], which not only provides joint lubrication and shock absorption and serves as a scaffold for the proteoglycans of the extracellular matrix (ECM) [27], but also supports cell growth and the chondrogenic differentiation of encapsulated stem cells, providing binding sites for growth factors [26].

HA, in particular, along with corticosteroids, represents the gold standard for the infiltrative treatment of knee OA, with a large number of randomized controlled trials now supporting its use, as opposed to the few studies present in the literature on other ECM proteins, such as collagen [28]. Conversely, there is not yet sufficient evidence that supports the use of PRP, although some studies have highlighted its efficacy [29].

The aim of this retrospective study was to compare the effectiveness of intra-articular injections of HA, PRP combined with HA (PRP-HA), and PBMNCs, in terms of the functioning of subjects affected by knee OA.

## 2. Materials and Methods

### 2.1. Participants

This retrospective observational study was conducted between January 2022 and July 2022 at the Pain Management Centre of the University Hospital of Catania, Italy. This study was carried out by anesthesiologists, in collaboration with residents from the Physical Medicine and Rehabilitation Department. Data were retrospectively analyzed in July 2023. Written informed consent was obtained from all participants, and this study conformed to the ethical guidelines of the 1975 Declaration of Helsinki. This study was approved by the Ethics Committee (Ethics Committee of Sicilia Region, n. 220—27 December 2023).

The subjects included in this study met the following criteria: (a) adults between 40 and 70 years old; (b) with normal prothrombin time (PT), partial thromboplastin time (PTT), and platelet count; (c) affected by unilateral symptomatic knee pain, with a history of chronic pain for at least 6 months; (d) unresponsive to conservative treatments, such as topical and oral medications as well as rehabilitation alone; (e) diagnosed as grade II-III according to the KL classification through knee X-ray imaging interpretation by a radiologist and a clinical physician. Subjects were excluded if they presented with any of the following: (a) local or systemic infection (septic arthritis), skin disorders, or knee effusion; (b) significant deformity of the knee or concurrent lesions (such as meniscal or ligamentous injuries or instability); (c) systemic diseases (including oncologic or hematologic diseases; severe renal, pulmonary, or hepatic impairment; and diabetes mellitus); (d) use of immunosuppressive drugs; (e) history of intra-articular treatment in the target knee within the past 6 months, or rehabilitation within the past 6 months; and (f) body mass index (BMI) greater than 30.5. None of the recruited patients had a history of alcohol abuse or routine tobacco use.

**Figure 1:**
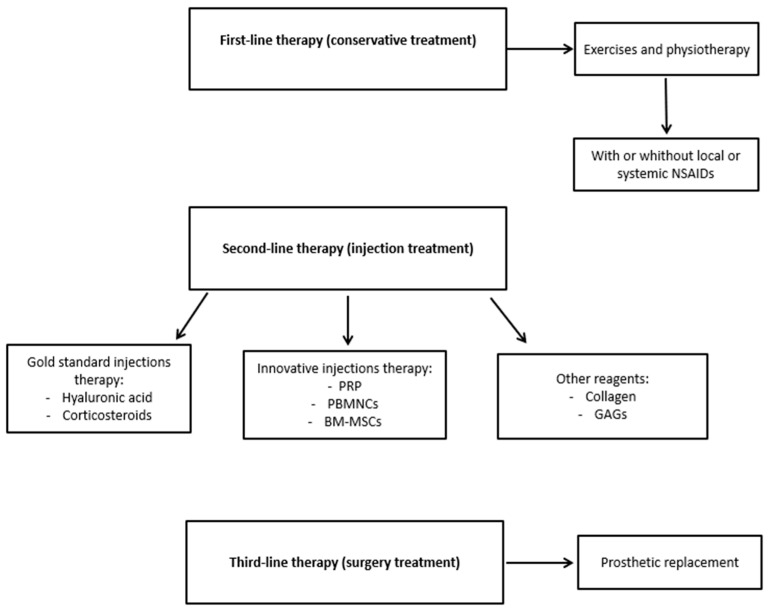
Schematic diagram of knee OA therapy. Abbreviations: non-steroidal anti-inflammatory drugs, NSAIDs; platelet-rich plasma, PRP; peripheral blood-derived mononuclear cells, PBMNCs; bone marrow-derived mesenchymal stem cells, BM-MSCs; glycosaminoglycans, GAGs.

The patients were unresponsive to first-line conservative treatment for knee OA and consequently a second-line therapy was proposed, as is the usual recommendation (see Figure 1).

The participants included in our study were divided into three groups: those receiving intra-articular injections of HA (2 weekly administrations of hybrid complexes of high- and low-molecular-weight HA (64 mg/2 mL)), PRP-HA (3 weekly intra-articular injections), and PBMNCs (1 injection of peripheral blood-derived mononuclear cells).

This study was conducted in a temperate climate region. Seasonal variations were present, with colder temperatures during the initial months, potentially influencing joint stiffness and pain perception. Participants resided in a mix of urban and suburban areas, with access to both paved and natural walking surfaces, which could affect joint load and mobility. In terms of dietary habits, most followed a Mediterranean-style diet, characterized by a high intake of vegetables, legumes, whole grains, and olive oil, with moderate consumption of fish and poultry. No specific dietary modifications were imposed during this study, but participants were encouraged to maintain their habitual eating patterns to minimize confounding factors related to nutrition. To support joint health and overall well-being, participants were advised to drink 1.5 to 2 L of water per day, as adequate hydration plays a crucial role in maintaining cartilage lubrication and metabolic functions.

### 2.2. Outcome Measures

The following data were analyzed at baseline and at one and six months following the final injection: (a) joint function assessed by the range of motion using a goniometer [30], (b) Numerical Rating Scale (NRS) [31], (c) Western Ontario and McMaster Universities Osteoarthritis Index (WOMAC) [32], (d) International Knee Documentation Committee (IKDC) score [33], (e) Timed Up and Go [34], (f) 10 m walking test, (g) Physical score (PCS12) and the Mental score (MCS12) on SF-12 for the quality of life [35], and (h) any adverse events were also recorded.

After 30 min of observation following the injection, the subjects were discharged with instructions to apply ice to the affected area and limit use of the leg for approximately 24 h, followed by gradual resumption of recreational activities.

### 2.3. Intervention

The medicated infiltration was performed in an operating room, ensuring sterile conditions of the environment and the affected area, in accordance with good clinical practice. Ultrasound-guided knee injections allowed for accuracy, precision, and repeatability [36,37].

We emphasize that high control practices were used during intra-articular injection therapy in order to avoid possible complications (joint infections, in particular) and to adequately inject the products inside the joint.

#### 2.3.1. Hyaluronic Acid Group

For the HA group, a commercially available HA was used, consisting of a buffered saline solution of hybrid complexes of high- and low-molecular-weight HA (32 mg high and 32 mg low for a total of 64 mg/2 mL), preserved in sterile conditions.

#### 2.3.2. Platelet-Rich Plasma Plus Hyaluronic Acid Group

**Figure 2:**
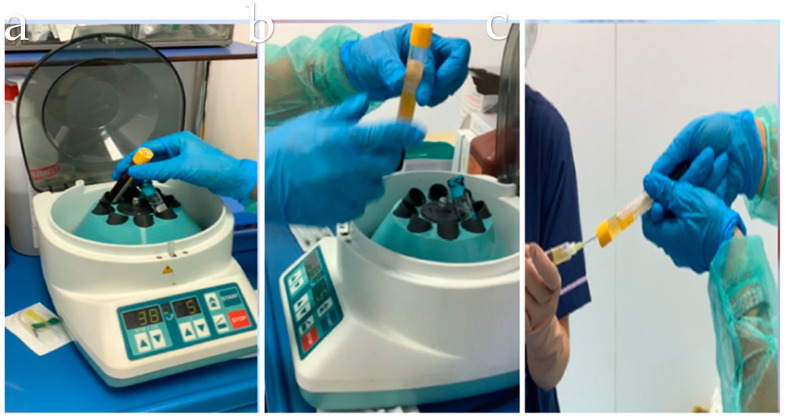
Preparation procedure for the intervention. (**a**) Blood collection with AI-filled tube; (**b**) centrifugation process; and (**c**) total 5 mL PRP + AI mixture.

For the PRP-HA group, a sample of 18 mL of peripheral venous blood was collected. Following centrifugation, in sterile conditions, to separate erythrocytes and concentrate platelets, 15 mL of PRP was obtained. A total of three doses of 5 mL each, one dose per week, of PRP and HA were prepared. The centrifugation parameters were as follows: (1) five-minute centrifugation, (2) 1500 g of centrifugal force, and (3) 32 rpm of velocity. After the centrifugation, the platelets and plasma were separated from other blood components and combined with the HA solution in the device. The resulting product was approximately 3 mL of autologous PRP, with a platelet concentration 1.5 to 1.6 times higher than the baseline blood level, and which contained a low number of red and white blood cells (resulting in neutrophil-poor PRP). The device also contained 2 mL of natural, non-cross-linked HA at a concentration of 20 mg/mL (totaling 40 mg). See Figure 2.

#### 2.3.3. Peripheral Blood-Derived Mononuclear Cells Group

For the PBMNCs group, a sample of 80–120 mL of peripheral blood was collected in a syringe containing 1 mL of heparin sodium 5000 UI/mL. This was processed and filtered, and a total of 60–120 mL of PBMNCs with regenerative potential were isolated. These aspirates were diluted in a 1:1 ratio (10 mL) with sterile saline solution. All procedures were performed in sterile conditions.

### 2.4. Statistical Analysis

R Statistical Software (4.4.3 Version) was used for data analysis. Continuous variables are presented as mean and standard deviation (SD). For comparisons both within and among groups, paired and unpaired Student’s *t*-tests were used, respectively. Analysis of variance (ANOVA) was performed to assess differences among groups for continuous, normally distributed, and homoscedastic data. With the unpaired Student’s *t*-test, this study compared the groups (HA vs. PRP-HA vs. PBMNCs) to identify the most effective treatment. Additionally, with the paired Student’s *t*-test, this study compared outcomes before and after injection to determine the trend of knee OA symptoms. For all tests, a *p*-value of less than 0.05 was considered statistically significant.

## 3. Results

This study included a total of 46 adults, 30 females and 16 males, with a mean age of 63.7 ± 10.9 years. These subjects, diagnosed with moderate-to-moderately severe knee OA (KL score: grades II-III), received intra-articular injections. Specifically, 14 subjects were treated with HA (HA group), 16 with PBMNCs (PBMNCs group), and 16 with PRP-HA (PRP-HA group).

The characteristics of this study’s sample are described in Table 1.

The groups were homogeneous for all the parameters except for age, which was significantly lower in the PBMNCs group compared to both the HA (*p* = 0.0041) and PRP-HA groups (*p* = 0.0006). Nine subjects (seven treated with HA and one with PRP-HA injections) were not included in the analysis due to incomplete data at the final evaluation.

The choice of treatment was influenced not only by the clinical needs, but also by the specific patient’s preferences. Indeed, some of the subjects declined the PBMNCs treatment to avoid blood manipulation. Similarly, others refused the PRP-HA, preferring HA, a treatment option with which they had fewer reservations. Furthermore, as HA has been more widely used and better known for a longer period, it tends to be more recognized, accepted, and requested by patients.

The PBMNCs group received a single intra-articular injection; the HA group was treated with two HA injections, once a week for two consecutive weeks; and the PRP-HA group was treated with three injections, once a week for three consecutive weeks. These protocols were based on different studies with similar reagents as used in ours [38,39,40].

Clinical results were obtained before the injection(s) and at the follow-ups, one and six months after the last injection.

All three treatments proved to be effective for (a) providing pain relief at one month (NRS: HA, *p* = 0.0072; PRP-HA, *p* = 0.0001; PBMNCs, *p* = 0.0169) and at six months post-injection (HA, *p* = 0.0118; PRP-HA, *p* = 0.0002; PBMNCs, *p* = 0.0098), and (b) improving mobility and reducing the risk of falls at one month (TUG: HA, *p* = 0.0026; PRP-HA, *p* = 0.0011; PBMNCs, *p* = 0.0394) and at six months post-injection (HA, *p* = 0.0449; PRP-HA, *p* = 0.0037; PBMNCs, *p* = 0.0147). Furthermore, significant improvements were observed in the WOMAC score at one month (HA, *p* = 0.0182; PRP-HA, *p* = 0.0017; PBMNCs, *p* = 0.0269), and at six months post-injection (HA, *p* = 0.0456; PRP-HA *p* = 0.0014; PBMNCs *p* = 0.0044). Specifically, in the first month, the WOMAC pain subscale improved significantly for the HA (*p* = 0.0093), PRP-HA (*p* = 0.0022), and PBMNCs (*p* = 0.0048) groups. This improvement was sustained at the six-month endpoint for the HA (*p* = 0.0050), PRP-HA (*p* = 0.0024), and PBMNCs (*p* = 0.0029) groups. However, the stiffness and physical activity domains proved the most difficult to influence in the first month for the HA (*p* = 0.1283; *p* = 0.0529) and PBMNCs (*p* = 0.4320; *p* = 0.0529) groups.

Additionally, the Physical Component Summary (PCS12) of the SF-12 showed a significant difference at both the first and sixth month after treatment with HA (at first month *p* = 0.0003; at sixth month *p* = 0.0036), PRP-HA (at first month *p* = 0.0005; at sixth month *p* < 0.0001), and PBMNCs (at first month *p* = 0.0009; at sixth month *p* = 0.0172). The Mental Component Summary (MCS12) of the SF-12 recorded significant improvements at the first month after a PRP-HA injection (*p* = 0.0298), and at the sixth month after a HA injection (*p* = 0.0409).

Furthermore, both PRP-HA and PBMNCs facilitated the recovery of a) ROM in flexion after one month (PRP-HA, *p* = 0.027; PBMNCs, *p* = 0.0169) and six months (PRP-HA, *p* = 0.0276; PBMNCs, *p* = 0.0009), and b) the IKDC score after one month (PRP-HA, *p* = 0.0001; PBMNCs, *p* = 0.0018) and six months post-injection (PRP-HA, *p* = 0.0001; PBMNCs, *p* = 0.0153).

Finally, PRP-HA also improved the ROM in extension after one month (*p* = 0.0128) and six months (*p* = 0.0128) post-injection.

Conversely, HA did not significantly improve the ROM, IKDC, or WOMAC stiffness. Both HA and PBMNCs did not effectively improve WOMAC stiffness or physical function at one month.

Furthermore, none of the three substances demonstrated significant effectiveness for improving the 10 MWT at one and six months, except the PBMNCs, which showed significant effects only in the first month.

**Figure 3:**
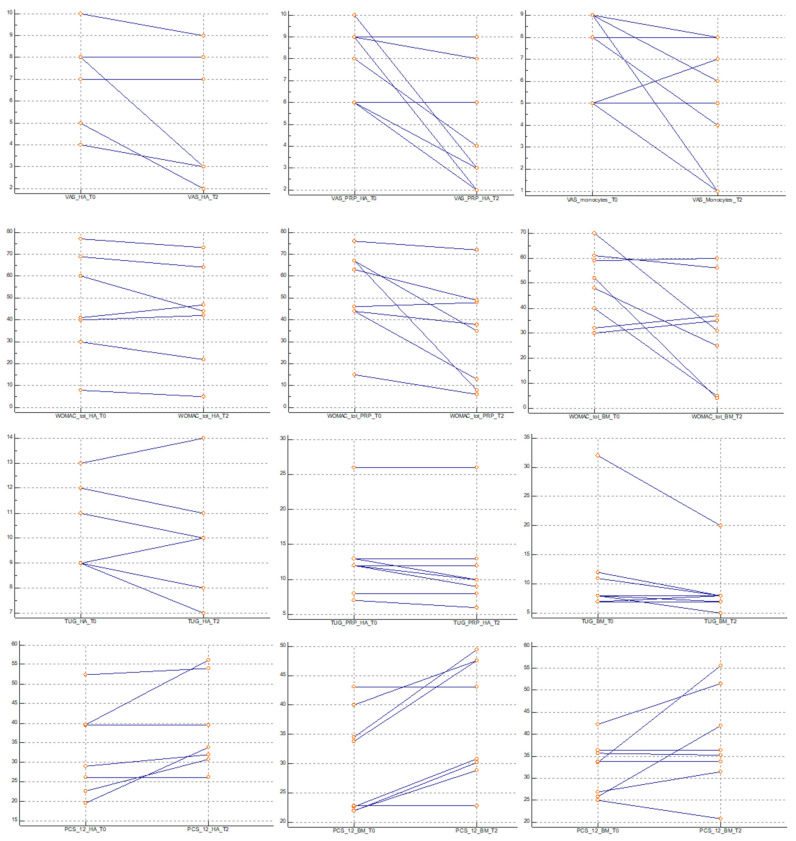
Dot-and-line diagram: NRS, WOMAC, TUG, and PACS-12 across the three groups.

Importantly, no serious adverse effects were observed in any of the three groups. A total of eight subjects experienced local reactions at the time of injection. After the HA treatment, one subject experienced mild pain (NRS = 3) in the injected joint within one hour of the injection, which required cold compression and several hours of rest. The pain disappeared within 24 h. After the PRP-HA treatment, four subjects developed moderate pain (NRS, 4.25 ± 0.50) and mild knee swelling four hours after the injection. Cold compression helped relieve the pain and swelling, which subsided within three hours. After the PBMNCs treatment, three subjects developed mild pain (NRS, 2.33 ± 0.58) within two hours post-injection, with spontaneous resolution in one subject, and the need for cold compression for relief in two subjects. No adverse effects related to the three types of injections were recorded at the one-month or six-month follow-up evaluations (see Table 2 and Figure 3 for further details).

**Figure 4:**
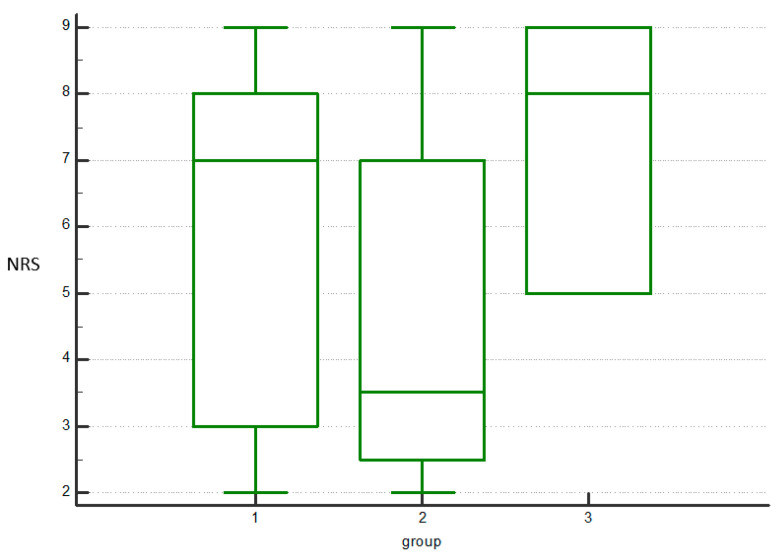
Representative multiple comparison graph of NRS at 6 months for the hyaluronic acid (1), PRP combined with hyaluronic acid (2), and peripheral blood-derived mononuclear cells groups (3), reflecting the absence of significant differences in all the other scales.

A comparison of the three groups revealed no significant difference in pain relief or functional recovery at one- and six-months post-injection across all the evaluated scales (see Table 3 and Figure 4).

## 4. Discussion

According to our knowledge, several studies exist in the field of intra-articular injection therapy. Many studies have compared the efficacy of HA to PRP, fewer have compared HA to BM-MSC, and according to our knowledge, no studies have analyzed all three therapies for the treatment of knee OA. For this reason, the aim of this study was to share the results of a comparison of HA, PRP-HA, and BM-MSC injections to better understand their potential for treating knee OA.

The biological role of injection therapies with HA, PRP, and PBMNCs has been studied and differs among the three substances. HA in its high-molecular-weight form has a replacement function and promotes the production of endogenous HA (viscosupplementation), while low-molecular-weight HA has an anti-inflammatory function and promotes the production of other endogenous HA (viscoinduction) [41]. PRP, on the contrary, with a large amount of growth factors and cytokines, has mainly reparative and anti-inflammatory functions [42], while PBMNCs, through the transformation of monocytes into macrophages, have anti-inflammatory, reparative, and regenerative functions [43].

The results of this study show that the HA, PRP-HA, and PBMNCs injections improved knee functional status, motor performance, and reduced pain at the follow-up, at one and six months after the last injection.

A meta-analysis compared the clinical effects of HA, steroids, PRP, and adipose mesenchymal stromal cell (MSC) injections for the treatment of knee OA [44]. Notably, for pain relief, steroids appeared to be the most effective treatment, followed by HA, while adipose MSCs and PRP seemed to be the least effective [44]. However, steroids offer temporary relief, and the benefits are often short-lived. Moreover, the PRP and adipose MSC interventions did not result in a significant reduction in joint pain or an improvement in joint function compared with a placebo. Additionally, steroids and HA were associated with a lower rate of adverse events compared to both the placebo and PRP [44]. However, the differences among the treatments were not clinically significant, indicating that other factors, such as cost and patient preferences, should be considered when treating patients with knee OA [44]. In fact, even in our study, some subjects showed a preference for one technique over the others, especially concerning their acceptance of blood manipulation or their familiarity and comfort level with a particular substance.

Several studies have shown no significant difference between PRP and HA in terms of functional recovery [45,46] and safety [47]. On the other hand, other studies have reported their effectiveness at reducing pain and improving knee function, with these results remaining stable over time, from 6 to 24 months [48]. Moreover, the median duration of subject-reported symptomatic relief was nine months for HA and 12 months for PRP. The only significant difference was observed in the reoperation rate at 24 months, which was significantly lower in the PRP group [6].

Additionally, low-molecular-weight HA, specifically, has seemed to yield similar positive results to PRP for improving knee function and relieving pain at two months, compared with high-molecular-weight HA [5,49]. This aspect could be attributed to the role of low-molecular-weight HA in restoring the rheological properties of joint synovial fluid (viscoinduction) [50]. For this reason, in this study, the HA group received a hybrid HA comprising complexes of both high- and low-molecular-weight HA, in order to combine the viscosupplementation effect associated with high-molecular-weight HA with the viscoinduction related to low-molecular-weight HA.

When comparing the effects of PRP with HA, several studies have reported significantly better results in terms of pain, function, stiffness, and quality of life after PRP injections compared to HA, at three, six, and twelve months [47,51,52]. The limited efficacy of HA compared to PRP [53] could be related to the severity of the knee OA. Nonetheless, the best results for PRP were documented for mild-to-moderate knee osteoarthritis [38].

Furthermore, PRP demonstrated superior results compared to HA in younger patients with cartilage lesions or early OA [5]. On the contrary, in geriatric patients, HA showed superior efficacy for reducing pain compared to PRP [54].

Lastly, a study demonstrated that even a sham control group, treated with saline solution injections, showed a statistically significant functional improvement from the baseline at the one-month follow-up. This phenomenon strongly suggested a positive placebo effect in the initial period following the injection [53].

The combination of PRP and HA has been shown to exert a synergistic effect for alleviating pain, improving joint function, reducing humoral and cellular immune responses, and promoting angiogenesis. Thus, the combined treatment has been shown to slow the progression of knee OA more effectively than PRP or HA treatment alone [38,55,56].

Moreover, their combined treatment has yielded stable and longer-term effects, up to 6 months [57]. Finally, it has been shown to be as safe as PRP injections alone [55,58]. Consistent with these findings, the PRP treatment proposed in this study also included HA to enhance its beneficial effects. However, the literature does not uniformly support these results. Indeed, according to other research, the combination of PRP with HA has not been proven to be superior to PRP alone for relieving pain and improving function in patients with knee OA [58,59].

In the past decade, mesenchymal stem cells (MSCs) have gained significant attention in the scientific community. Many studies have demonstrated their effectiveness at enhancing clinical outcomes, such as pain relief, functional recovery, and cartilage repair, in patients with osteoarthritis (OA) [60,61].

PBMNCs have been shown to have an anti-inflammatory effect comparable to that of bone marrow-derived mesenchymal stem cells [62]. The intra-articular injection of PBMNCs has been deemed safe, with no severe side effects observed over a 12-month period [63].

Administering moderate-to-high numbers of cells (40 × 10^6^) has been found to yield optimal responses in individuals with a knee OA grade ≥2 on the Kellgren–Lawrence (KL) classification. The use of a larger number of cells (100 × 10^6^) was associated with better improvements in knee OA, although with higher risks of adverse events [4].

In patients with moderate-to-advanced knee OA (grades II-IV on the KL scale), a single intra-articular PBMNCs injection resulted in significant improvements in pain intensity and the quality of the articular cartilage. These improvements persisted from 12- [40,64] to 24-months post-injection [62,65]. In the case of mild-to-moderate knee OA (KL stages II-III), a PBMNCs injection yielded clinical improvements in pain, function, and quality of life. These improvements began to decline after 6 months [66], but the patients still reported feeling better for an additional 12 [67,68] to 60 months compared to the baseline [66].

When compared to the HA treatment, mononuclear cells offered significant clinical improvement, better pain relief, and the long-term functional enhancement of the knee (over 12 months) [63]. This appears to be linked to the ability of PBMNCs to slow the degenerative structural changes in the knee joint tissue [69].

It is particularly interesting that this work analyzed data after the first COVID-19 pandemic, taking into account hypothesis of potential risk of developing KOA during this phase, as reported by clinical and epidemiological analyses on the rehabilitation need after COVID-19 with a consequent improvement of home-based rehabilitation [70,71,72,73,74,75,76].

In conclusion, we are aware that this study had some limitation, involving a relatively small number of participants (considering to the study design) and a relatively short follow-up period of six months. A larger sample size, encompassing a broader range of ages and OA severities, would allow for a more detailed stratification of the results. Nonetheless, the inconsistencies in the current literature on this topic, coupled with the absence of definitive guidelines, highlight the importance of sharing of our experience and the protocol we utilized for knee OA injections.

However, to the best of our knowledge, this is the first study to compare the effects of HA, PRP-HA, and PBMNCs injections for the treatment of OA.

## 5. Conclusions

Taken together, the findings of this retrospective study show that injections of HA, PRP-HA, and PBMNCs demonstrated positive outcomes for up to 6 months post-treatment in subjects suffering from knee OA. However, no significant differences were observed among these three treatment modalities in terms of pain reduction, functional recovery, or quality of life improvement. In conclusion, all three types of injections demonstrated nearly equal outcomes in terms of pain relief, mobility improvement, fall risk reduction, stiffness reduction, and functional enhancement.

The current literature on OA presents substantial variability in the treatment selection, dosage, and follow-up duration. This variability has hindered the development of standardized guidelines, and the identification of the most effective treatment based on OA severity and patient-specific characteristics.

Therefore, additional clinical trials are necessary to determine the most effective treatment based on both the overall and patient-specific clinical characteristics. A standardized protocol is needed to guide physicians in selecting the most appropriate injectable treatment for knee OA.

## Figures and Tables

**Table 1:** Characteristics of this study’s participants.

Participants	Hyaluronic Acid	PRP-HA	Monocytes
**Number of subjects**	14	16	16
**Age**	66.4 ± 4.03	69.8 ± 8.08	55.4 ± 12.7
**m/f**	4/10	4/12	8/8
**KL grade II**	8	9	9
**KL grade III**	6	7	7
**HA group**			
	Before injection	After 1 month	After 6 months
**Knee ROM in extension**	2.86 ± 4.69	1.43 ± 3.63	1.43 ± 3.63
**Knee ROM in flexion**	113.57 ± 12.62	115.71 ± 10.89	115.71 ± 10.89
**NRS**	7.14 ± 1.95	5.14 ± 2.68	5.71 ± 2.81
**IKDC**	35.14 ± 20.33	43.75 ± 18.96	45.23 ± 23.00
**WOMAC pain**	11.28 ± 4.59	8.86 ± 5.30	9.28 ± 5.45
**WOMAC stiffness**	2.43 ± 2.14	2.14 ± 2.31	2.28 ± 2.27
**WOMAC physical function**	32.71 ± 16.94	30.71 ± 14.87	30.85 ± 15.31
**Womac total**	46.43 ± 23.02	41.71 ± 21.72	42.43 ± 22.33
**TUG**	10.28 ± 1.64	9.43 ± 2.06	9.57 ± 2.41
**10MWT**	9.14 ± 2.17	8.85 ± 2.63	8.71 ± 2.98
**PCS-12**	32.70 ± 11.13	38.97 ± 9.59	38.92 ± 11.25
**MCS-12**	43.75 ± 7.85	46.49 ± 5.86	46.79 ± 6.47
**Adverse events**	-	1 pt local reaction	-
**PRP-HA group**			
	Before injection	After 1 month	After 6 months
**ROM in extension**	3.75 ± 5	0.63 ± 1.71	121.9 ± 12.63
**ROM in flexion**	115.62 ± 9.46	122 ± 12.6	0 ± 0
**NRS**	7.88 ± 1.59	4 ± 2.53	4.63 ± 2.63
**IKDC**	31.46 ± 19.78	58.8 ± 21.3	56.9 ± 19.4
**WOMAC pain**	9.75 ± 4.02	5.38 ± 4.81	6 ± 4.35
**WOMAC stiffness**	2.25 ± 1.69	1.25 ± 1.34	1.5 ± 1.26
**WOMAC physical function**	40.8 ± 15	24.8 ± 18.8	26.12 ± 17.6
**WOMAC total**	52.3 ± 18.3	31 ± 23.3	33.62 ± 22.45
**TUG**	12.9 ± 5.57	11.6 ± 5.95	11.75 ± 5.95
**10MWT**	10.30 ± 2.62	9.5 ± 2.25	9.5 ± 2.25
**PCS-12**	30.1 ± 8.49	36.6 ± 9.44	37.56 ± 10.09
**MCS-12**	42.3 ± 8.76	46 ± 6.89	44.20 ± 6.27
**Adverse events**	-	4 pt local reaction	-
**PBMNCs group**			
	Before injection	After 1 month	After 6 months
**ROM in extension**	0.62 ± 1.71	0 ± 0	0 ± 0
**ROM in flexion**	113.12 ± 14.13	122.5 ± 8.94	121.87 ± 8.14
**NRS**	7.25 ± 1.84	5.62 ± 2.53	5.00 ± 2.73
**IKDC**	32.36 ± 15.02	51.69 ± 20.43	54.65 ± 22.40
**WOMAC pain**	11.62 ± 4.58	8 ± 4.32	6.25 ± 4.58
**WOMAC stiffness**	1.87 ± 1.5	1.62 ± 1.62	1 ± 1.46
**WOMAC physical function**	35.5 ± 10.11	29 ± 17.16	24.37 ± 14. 33
**WOMAC total**	49.00 ± 13.79	38.62 ± 22.18	31.62 ± 19.85
**TUG**	11.62 ± 8.15	8.62 ± 2.92	8.87 ± 4.45
**10MWT**	9.37 ± 2.92	7.87 ± 1.50	8.50 ± 3.83
**PCS-12**	32.45 ± 5.85	38.50 ± 9.11	38.37 ± 10.77
**MCS-12**	46.93 ± 8.54	46.25 ± 10.36	44.61 ± 10.08
**Adverse events**	-	3 pt local reaction	-

Abbreviations: Kellgren and Lawrence, KL; male, m; female, f; range of motion, ROM; Numerical Rating Scale, NRS; hyaluronic acid, HA; platelet-rich plasma combined with hyaluronic acid, PRP-HA; peripheral blood-derived mononuclear cells, PBMNCs; month, m; Western Ontario and McMaster Universities Osteoarthritis Index, WOMAC; International Knee Documentation Committee, IKDC; Timed Up and Go, TUG; 10 meter walking test, 10MWT; Patients pt; Physical score PCS-12; Mental score MCS-12.

**Table 2:** Comparison among HA, PRP-HA, and PBMNCs group. Results after one month (T1) and 6 months (T6) from the last injection, compared to T0 (before injections).

Scores	Injection	Paired *t*-Test T0–T1	Paired *t*-Test T0–T6
**ROM flexion**	HA	*p* = 0.0537	*p* = 0.0537
	PRP-HA	*p* = 0.0276	*p* = 0.0276
	BM	*p* = 0.0169	*p* = 0.0009
**ROM extension**	HA	*p* = 0.1648	*p* = 0.1648
	PRP-HA	*p* = 0.0128	*p* = 0.0128
	BM	*p* = 0.1639	*p* = 0.1639
**NRS**	HA	*p* = 0.0072	*p* = 0.0118
	PRP-HA	*p* = 0.0001	*p* = 0.0002
	BM	*p* = 0.0169	*p* = 0.0098
**IKDC**	HA	*p* = 0.0642	*p* = 0.1253
	PRP-HA	*p* = 0.0001	*p* = 0.0001
	BM	*p* = 0.0018	*p* = 0.0153
**WOMAC total**	HA	*p* = 0.0182	*p* = 0.0456
	PRP-HA	*p* = 0.0017	*p* = 0.0014
	BM	*p* = 0.0269	*p* = 0.0044
**WOMAC pain**	HA	*p* = 0.0093	*p* = 0.0050
	PRP-HA	*p* = 0.0022	*p* = 0.0024
	BM	*p* = 0.0048	*p* = 0.0029
**WOMAC stiffness**	HA	*p* = 0.1648	*p* = 0.1648
	PRP-HA	*p* = 0.0152	*p* = 0.0032
	BM	*p* = 0.4320	*p* = 0.0252
**WOMAC physical function**	HA	*p* = 0.1283	*p* = 0.0080
	PRP-HA	*p* = 0.0021	*p* = 0.0017
	BM	*p* = 0.0529	*p* = 0.0080
**TUG**	HA	*p* = 0.0026	*p* = 0.0449
	PRP-HA	*p* = 0.0011	*p* = 0.0037
	BM	*p* = 0.0394	*p* = 0.0147
**10MWT**	HA	*p* = 0.3909	*p* = 0.1648
	PRP-HA	*p* = 0.1108	*p* = 0.1108
	BM	*p* = 0.0032	*p* = 0.1546
**PCS-12**	HA	*p* = 0.0003	*p* = 0.0036
	PRP-HA	*p* = 0.0005	*p* < 0.0001
	BM	*p* = 0.0009	*p* = 0.0172
**MCS-12**	HA	*p* = 0.0818	*p* = 0.0409
	PRP-HA	*p* = 0.0298	*p* = 0.1433
	BM	*p* = 0.6607	*p* = 0.2323

Abbreviations: range of motion, ROM; Numerical Rating Scale, NRS; hyaluronic acid, HA; platelet-rich plasma combined with hyaluronic acid, PRP-HA; peripheral blood-derived mononuclear cells, PBMNCs; month, m; Western Ontario and McMaster Universities Osteoarthritis Index, WOMAC; International Knee Documentation Committee, IKDC; Timed Up and Go, TUG; 10 meter walking test, 10MWT; Physical score, PCS-12; Mental score, MCS-12.

**Table 3:** Comparison among HA group, PMR group, and PBMNCs group with ANOVA.

Differences Among the Groups	F-Ratio at T0	Significance Level at T0	F-Ratio at T1	Significance Level at T1	F-Ratio at T6	Significance Level at T6
NRS	0.752	*p* = 0.478	1.671	*p* = 0.200	0.610	*p* = 0.548
ROM knee extension	2.518	*p* = 0.092	1.524	*p* = 0.229	1.602	*p* = 0.213
ROM knee flexion	0.189	*p* = 0.829	1.721	*p* = 0.191	2.271	*p* = 0.056
IKDC	0.159	*p* = 0.854	2.039	*p* = 0.143	1.207	*p* = 0.309
WOMAC total	0.373	*p* = 0.691	0.927	*p* = 0.404	1.043	*p* = 0.361
WOMAC pain	0.815	*p* = 0.449	0.421	*p* = 0.659	2.133	*p* = 0.131
WOMAC stiffness	0.384	*p* = 0.684	0.941	*p* = 0.398	2.178	*p* = 0.126
WOMAC physical function	1.259	*p* = 0.294	0.492	*p* = 0.615	0.429	*p* = 0.654
TUG	1.186	*p* = 0.315	2.307	*p* = 0.112	1.700	*p* = 0.195
10_MWT	0.771	*p* = 0.469	2.306	*p* = 0.111	0.460	*p* = 0.634
PCS-12	0.421	*p* = 0.659	0.275	*p* = 0.761	0.0627	*p* = 0.939
MCS-12	1.285	*p* = 0.287	0.0168	*p* = 0.983	0.459	*p* = 0.635

Abbreviations: range of motion, ROM; Numerical Rating Scale, NRS; hyaluronic acid, HA; platelet-rich plasma combined with hyaluronic acid, PRP-HA; month, m; Western Ontario and McMaster Universities Osteoarthritis Index, WOMAC; International Knee Documentation Committee, IKDC; Timed Up and Go, TUG; 10 meter walking test, 10MWT; Physical score, PCS-12; Mental score, MCS-12; peripheral blood-derived mononuclear cells, PBMNCs.

## Data Availability

The dataset is available on request from the corresponding author.
